# Leaking Bleb After 50 Years of Historical Iridencleisis Surgery: A Case Report

**DOI:** 10.7759/cureus.104869

**Published:** 2026-03-08

**Authors:** Shunsuke Nakakura, Ryota Aoki, Santaro Noguchi, HIroyuki Wakuda, Shinichiro Kuroda

**Affiliations:** 1 Department of Ophthalmology, Saneikai Tsukazaki Hospital, Himeji, JPN; 2 Department of Ophthalmology, Nagata Eye Clinic, Nara, JPN

**Keywords:** autograft scleral flap, glaucoma, iridencleisis, leaking bleb, trabeculectomy

## Abstract

We describe a case of historical iridencleisis surgery in a patient who initially presented to our hospital with leaking blebs, presumed to be secondary to trabeculectomy. The patient was a 70-year-old male with a history of an uncertain glaucoma surgery performed on his left eye at 18 years of age (1973). At presentation, his intraocular pressure (IOP) was 3 mmHg and visual acuity was hand motion. Examination revealed a positive Seidel test from a diffuse, large ischemic bleb with a large iridectomy. Given a history of two prior episodes of blebitis, we planned bleb reconstruction surgery. Intraoperatively, we discovered invaginated iris tissue without a scleral flap, a finding distinct from conventional trabeculectomy. We created a rectangular, half-thickness autograft scleral flap from an adjacent site and used it to cover the invaginated iris, thereby stopping the aqueous humor leakage. These intraoperative features confirmed that the original procedure was iridencleisis, a historical surgical technique developed by Søren Holth in 1907. This procedure was widely used until the 1950s but subsequently declined due to the risk of sympathetic ophthalmia and insufficient safety and predictability. Postoperatively, aqueous humor leakage ceased and IOP increased. Although severe visual field defects persisted, the patient's visual acuity improved to 0.7 (logMAR) following cataract surgery, and IOP remained stable at approximately 14 mmHg. We report a rare case of historical iridencleisis surgery, noting that its blebs resembled those of trabeculectomy despite the absence of antimetabolite use.

## Introduction

Glaucoma surgery has a long history, originating from iridectomy performed by Albrecht von Graefe in 1856 [1]. For filtering surgery, De Wecker introduced sclerotomy as a procedure for chronic glaucoma in 1858. Additionally, in 1900, internal filtration (cyclodialysis) was developed [1]. Modern trabeculectomy was first reported by Cairns [2] in 1968 and was further refined through the application of mitomycin-C and 5-FU in the 1980s [3,4]. Throughout the historical trajectory of glaucoma surgery, many once-promising techniques have eventually been superseded by more modern interventions. However, between 1900 and 1968, another filtering surgery technique called "iridencleisis" existed, which was reported by Holth in 1909 [5,6] among the earlier full-thickness fistulas such as sclerectomy, trephination, and thermal sclerostomy (Scheie procedure) [7]. This technique was modified [8]; however, it was eventually superseded by the more modern trabeculectomy. Herein, we report a case of this historical iridencleisis surgery that presented with a modern trabeculectomy-like bleb, which was identified incidentally during bleb reconstruction surgery. This study was approved by the Institutional Review Board of Saneikai Tsukazaki Hospital, Himeji, Japan (No: 2508002).

## Case presentation

**Figure 1:**
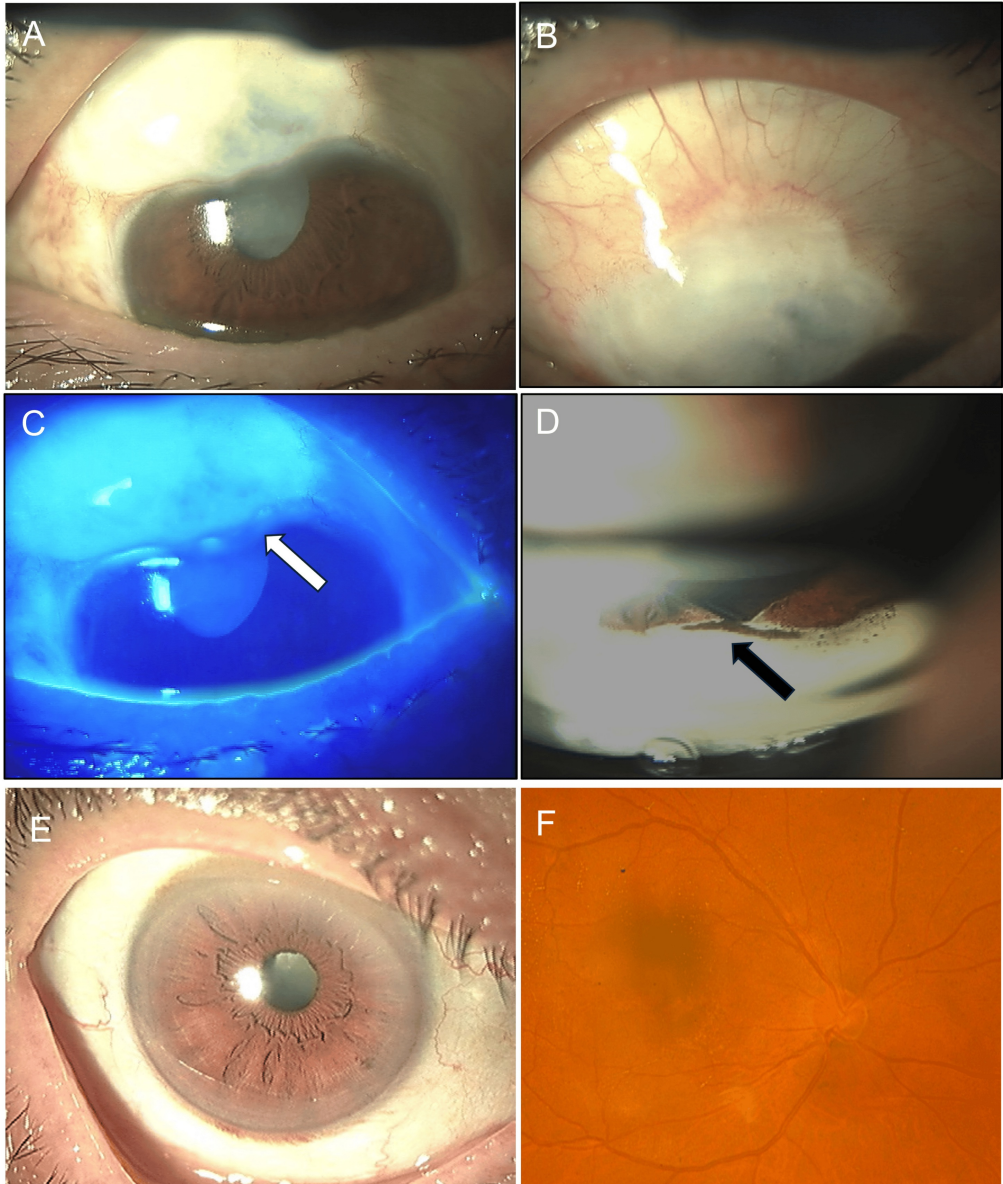
Preoperative Clinical Findings A, B. Large ischemic bleb and mature cataract. C. Aqueous humor leakage near the corneal limbus. D. Gonioscopic view showing iris incarceration into the filtering window. E. Senile cataract, right eye. F. The optic disc showed no glaucomatous change, right eye.

A 70-year-old male was referred to our department for a leaking bleb, with a history of glaucoma surgery of uncertain type performed on his left eye at 18 years of age (1973). The patient was interviewed regarding his medication history and the specific glaucoma diagnosis from 50 years ago; however, he was unable to recall the details due to the extensive time elapsed. At presentation, his intraocular pressure (IOP) was 3 mmHg by Goldmann applanation tonometer, and visual acuity was hand motion in the left eye. Examination revealed a positive Seidel test from a diffuse, large ischemic bleb. A large iridectomy, pupil deviation, and mature cataract were also observed (Figure 1A-C). Gonioscopic examination demonstrated incursion of iris tissue into the filtering window (Figure 1D). The retinal status could not be evaluated by ophthalmoscopy due to the mature cataract. Corneal endothelial cell density was 2,165 cells/mm².

The patient had experienced two prior episodes of blebitis treated medically. Examination of the right eye revealed a cataract; however, both gonioscopic and funduscopic findings were unremarkable (Figure 1E and F). His IOP in the right eye was 14 mmHg by Goldmann applanation tonometer, and visual acuity was 0.1 (logMAR) in the right eye. Given the surgical history and recurrent blebitis, we planned bleb reconstruction surgery to cease aqueous humor leakage and restore a functional filtering bleb.

Surgical procedure

**Figure 2:**
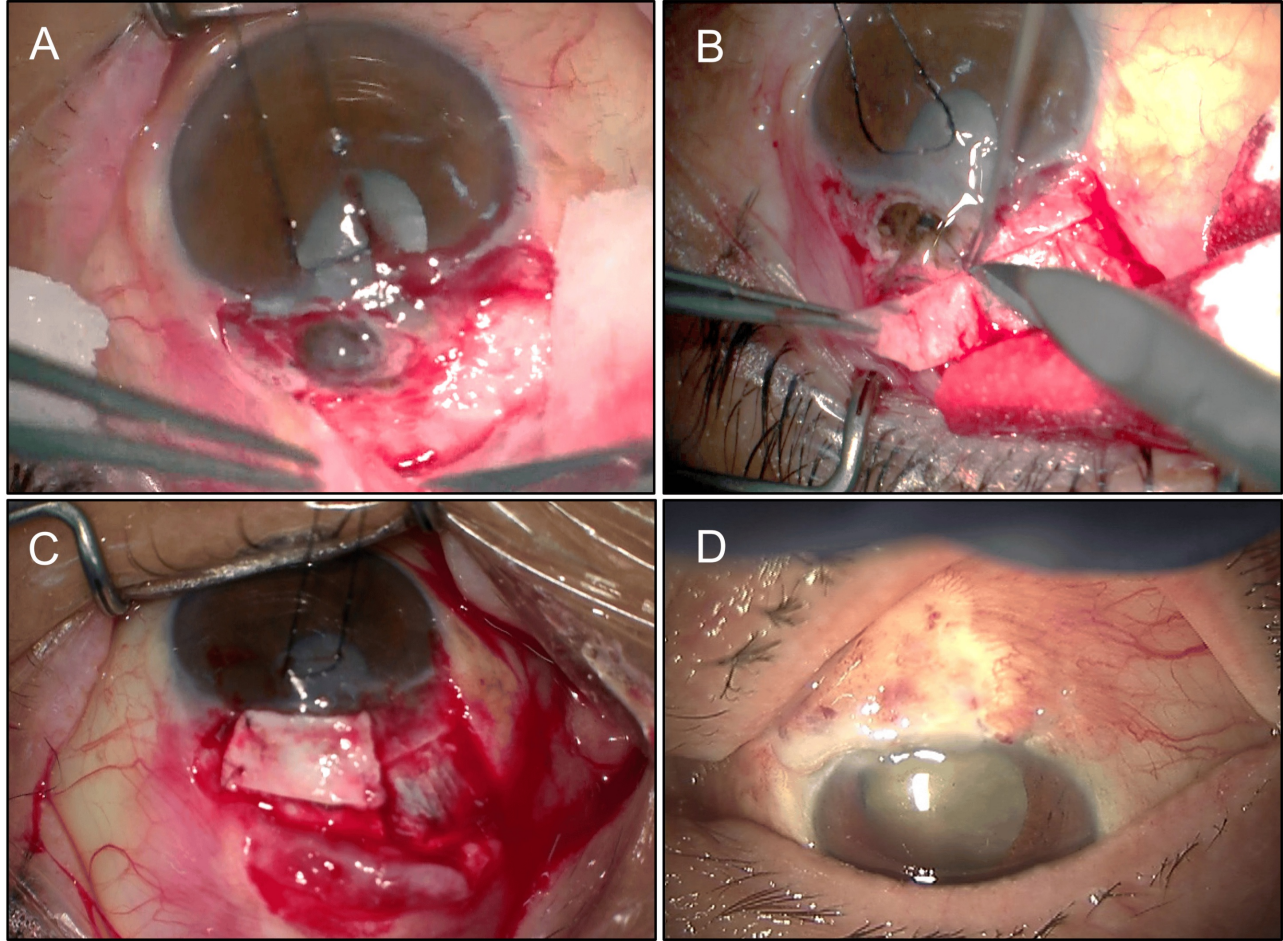
Intraoperative Findings and Surgical Technique A. No scleral flap from trabeculectomy was identified; instead, incarcerated iris tissue covered by thin fibrous tissue with active aqueous humor leakage was observed. B. Harvesting of a rectangular, half-thickness autologous scleral flap from an adjacent site. C. The scleral graft secured in place with sutures. D. Postoperative appearance demonstrating absence of aqueous humor leakage.

Following topical and sub-Tenon's anesthesia, the ischemic conjunctiva was opened. Intraoperatively, no scleral flap from trabeculectomy was identified; instead, we found incarcerated iris tissue covered by thin fibrous tissue with active aqueous humor leakage (Figure 2A). This surgical anatomy was unprecedented in our experience, and creation of a new scleral flap around the incarcerated iris tissue appeared unfeasible due to the large fistula. Therefore, we elected to close the fistula using a covering technique. A rectangular, half-thickness autologous scleral flap was harvested from an adjacent site and used to cover the area of iris incarceration (Figure 2B). The scleral graft and conjunctiva were sutured with 8-0 Vicryl suture (Figure 2C). On postoperative day one, IOP increased to 8 mmHg without aqueous humor leakage.

Clinical course

**Figure 3:**
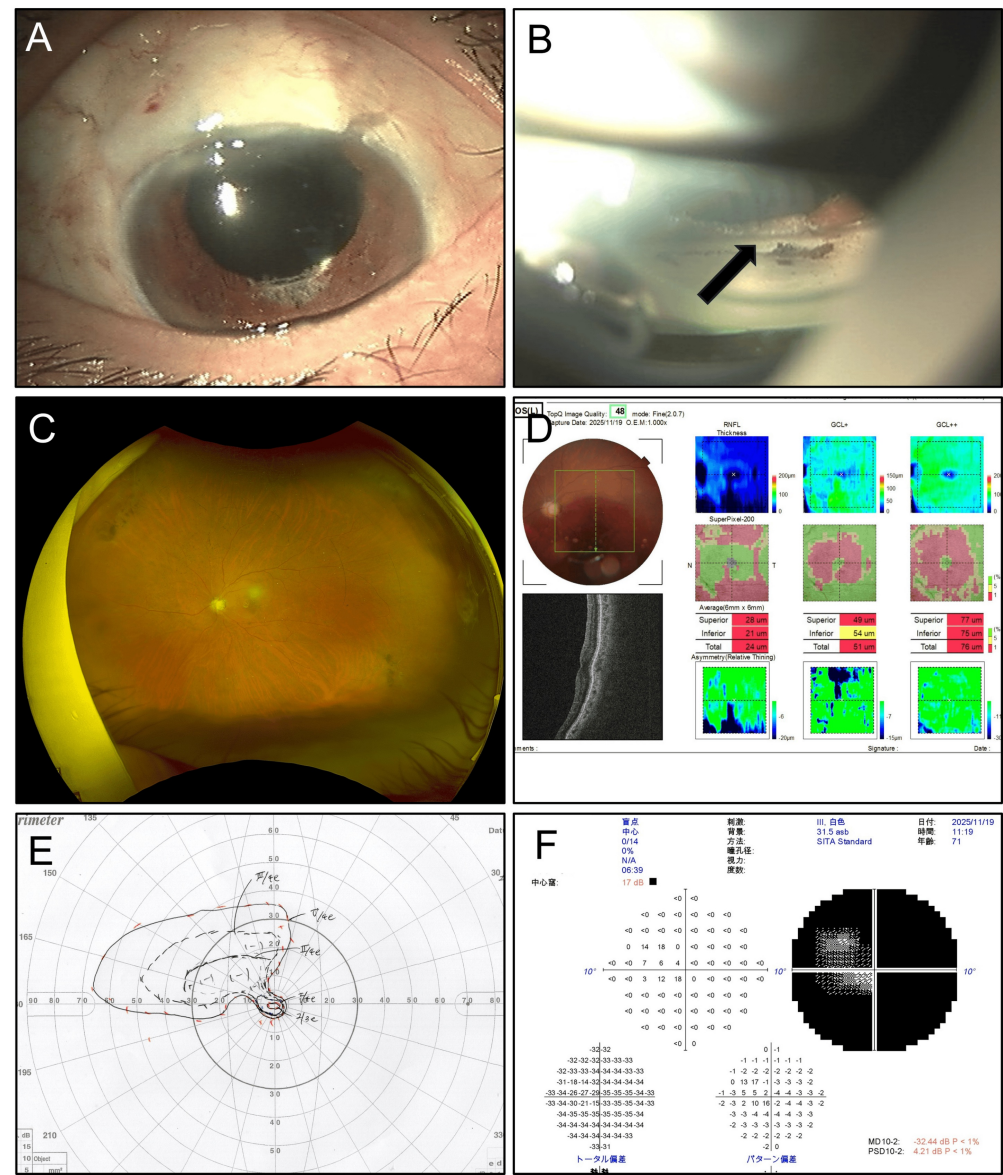
Postoperative Clinical Findings A. Anterior segment appearance following cataract surgery. B. Iris incarceration released during cataract surgery. C. Fundus photograph showing optic disc pallor consistent with optic atrophy. D. Optical Coherence Tomography. E. Severe visual field defect (Aulhorn stage IV ). F. Mean deviation -32.44 dB by Humphrey Field Analyzer 10-2 program.

One month following the initial surgery, the patient underwent phacoemulsification with intraocular lens implantation (Figure 3A). During this procedure, the incarcerated iris was released from the filtering window to attempt iris reconstruction to address both functional and cosmetic concerns (Figure 3B). Postoperative retinal imaging revealed simple optic disc atrophy (Figure 3C). Optical coherence tomography also showed a pale disc and thinning of the retinal layers (Figure 3D). Additionally, brain magnetic resonance imaging (MRI) was performed to exclude optic nerve tumors or optic neuritis; no abnormalities were detected. Goldmann perimetry (Figure 3E) and Humphrey Field Analyzer 10-2 program (Figure 3F) demonstrated severe visual field defects. At six months following the initial presentation, left eye visual acuity had recovered to 0.7 (logMAR), and IOP remained stable at approximately 14 mmHg with topical carteolol hydrochloride 2%.

## Discussion

Initially, we suspected a leaking bleb following modern trabeculectomy with adjunctive antimetabolite application. However, intraoperative findings revealed an unconventional surgical footprint. Upon consultation with an experienced co-author (S.K.), it was hypothesized that the procedure was a historical iridencleisis. Our intraoperative observations, specifically the absence of a scleral flap, incarceration of the iris within the scleral stoma (Figure 2), and localized iridodialysis (Figure 1D), align with the surgical techniques characterized by Reese (1958) [9]. This historical procedure primarily involved radial iris incision and permanent iris incarceration into the sclera to facilitate aqueous humor filtration [9].

While Scheie’s sclerectomy (developed in the 1950s) was considered as a differential surgical option, it is distinguished by the absence of iris incarceration [10]. In a 1959 study of 95 consecutive patients, Haisten et al. reported a complete success rate (IOP < 23 mmHg, stable visual fields, and intact surgical sites) of 75%, which increased to 84% with adjunctive miotics [11]. Common early complications included hyphema (30%) and a shallow or flat anterior chamber (26%) [11].

Despite these results, iridencleisis was eventually superseded by modern trabeculectomy. Cvetkovic et al. (1979) demonstrated that trepanotrabeculectomy offered superior IOP control and a lower complication profile compared with iridencleisis [12]. While Gess et al. (1985) proposed a modified technique involving iris incarceration under a scleral flap [13], the advent of 5-Fluorouracil and Mitomycin C in the 1980s significantly enhanced the predictability and success rates of trabeculectomy [14,15] by excising uveal tissue [16].

Given the normal status of the right eye, the left eye was presumed to have unilateral secondary glaucoma. Nevertheless, it is difficult to ascertain the surgical intent, considering how much the standard of care has changed over the intervening 50 years.

## Conclusions

We report a rare case of historical iridencleisis featuring a filtering bleb that closely resembled those seen in modern trabeculectomy. When clinicians encounter a large ischemic bleb associated with a prominent surgical iridectomy, iridencleisis should be considered in the differential diagnosis of prior glaucoma surgery.
